# Chemsex Session Typologies and Associated Sociodemographic Factors in Sexual Minority Men: Latent Class Analysis From a Cultural Perspective Using a Cross-Sectional Survey

**DOI:** 10.2196/60012

**Published:** 2024-09-27

**Authors:** Paule Gonzalez-Recio, Rose Crossin, Marta Donat, David Palma, David Guede Caballero, Sara Moreno-Garcia, Juan Miguel Guerras, María José Belza

**Affiliations:** 1 National Health School, Carlos III Health Institute, Av Monforte de Lemos 5, Madrid, 28029, Spain, 34 918222196; 2 Center for Biomedical Research Network in Epidemiology and Public Health, Madrid, Spain; 3 Department of Population Health, University of Otago, Christchurch, New Zealand; 4 Department of Epidemiology, Barcelona Public Health Agency, Barcelona, Spain; 5 Department of Preventive Medicine, Cruces University Hospital, Barakaldo, Spain; 6 Department of Preventive Medicine, Severo Ochoa University Hospital, Leganés, Spain

**Keywords:** chemsex, sexualized drug use, sexual minority men, typologies, latent class analysis, social determinants of health, culture, party and play, sociodemographics, sexual minority, cross-sectional study, homogeneity, SMM, Spain, drug use, Poisson regression, migrants, public health, LGBTQ, teenagers, adults, HIV, sexual health, sexual risk behavior, gay

## Abstract

**Background:**

Chemsex prevalence is still not well known, and both the lack of homogeneity and cultural component of chemsex practices are usually overlooked.

**Objective:**

This study aims to estimate the proportion of sexual minority men (SMM) engaging in chemsex sessions, while understanding the cultural dimension of chemsex, and to analyze distinct session typologies with potential risk differences and the sociodemographic factors associated with engaging in them.

**Methods:**

A total of 5711 SMM residing throughout Spain participated in an anonymous web-based survey that assessed chemsex session engagement and characteristics, drug use, and sociodemographic variables. We measured the association of sociodemographic factors with engaging in chemsex sessions by calculating adjusted prevalence ratios, using multivariate Poisson regression analysis. Chemsex typologies were analyzed using latent class analysis, and sociodemographic factors were associated with the different risk classes.

**Results:**

Our results determined that 21.1% (1205/5711; 95% CI 20.0%‐22.1%) of SMM engaged in chemsex sessions during their lifetime. Participating in sessions was significantly associated with being a migrant, not having a comfortable financial situation, openly living their sexuality, residing in bigger municipalities, older age, using steroids, and living with HIV (adjusted prevalence ratio: range 1.17-2.01; all *P* values <.05). Three typologies of sessions with different risks were identified with latent class analysis, with 23.2% of SMM engaging in sessions taking part in higher-risk ones, which was associated with younger age, using steroids, living in bigger municipalities, openly living their sexuality, and living with HIV, compared to SMM engaging in lower-risk sessions (odds ratio: range 2.75-4.99).

**Conclusions:**

Chemsex is relatively common among SMM in Spain, but it is important to differentiate typologies of sessions with varying risks, and the proportion of SMM engaging in high-risk sessions is low. Chemsex is highly associated with sociodemographic factors. Chemsex should be prioritized in public health programs, which should consider the different forms of sessions with their varying risks and prevalence, while also considering the cultural dimension inherent to chemsex.

## Introduction

Over the last two decades, chemsex has become more prevalent within the LGBTQAI+ community, particularly among cis sexual minority men (SMM). Chemsex is typically defined as a specific type of sexualized substance use in the queer community [1], within which it has emerged as a cultural phenomenon [2,3]. There are several substances that are particularly associated with chemsex or sexualized use, such as gamma-hydroxybutyrate (GHB), methamphetamine, mephedrone, ketamine, and poppers, although the use of these substances is not exclusive to chemsex [1]. The cultural aspect of chemsex is key to its definition, as it is largely shaped by shared experiences of discrimination and the role of masculinity and body image pressure within a subset of the community [4,5]. People who participate in chemsex have created a specific scene, codes, and vocabulary around it [6,7], which also reinforces its cultural component. Chemsex sessions—events or gatherings for this type of sexualized drug use—are a fundamental part of this culture and may also be referred to as “party and play” or “chill,” depending on the context.

This sexual cultural phenomenon has gained significance within public health, as there are risks associated with substance use (including the criminalization of the substances) and certain sexual activities [8-10]. Notably, the injection of substances as part of chemsex—termed “slamming”—presents a particular risk due to the higher risk associated with this consumption route as well as the risk of transmission of certain infections or injecting-related injuries like abscesses. However, chemsex remains a low priority in most public health and research institutions, with health promotion programs depending on LGBTQAI+ community–based organizations [11].

The prevalence of chemsex is still not well known. Most studies estimating the percentage of SMM engaging in chemsex have significant limitations, such as including only SMM living with HIV [12-14], recruiting participants from sexually transmitted infection clinics (where high-risk practices are overrepresented) [15-20], having small sample sizes [12,13,15,16,20], or identifying any use of certain substances as chemsex [13,14,18,19,21]. Furthermore, despite sessions being a fundamental aspect of the cultural concept of chemsex, the vast majority of studies define any sexualized substance use as chemsex, disregarding specific contexts, participation in chemsex sessions, or participants’ referring to what they do as chemsex [15-17,20,22-24].

Previous articles have found that chemsex is associated with different sociodemographic factors [23,25-29]. Some of those factors are age [25-29], living with HIV [23,25,28,29], living in larger cities [25], ethnic heritage [27], migratory status [29,30], educational level [25], financial status [30], or openly living their sexuality [25]. However, these studies had the same limitation as those that investigated the proportion of SMM engaging in chemsex, as they classified any use of certain substances as chemsex.

Evidence shows that chemsex is not a homogeneous phenomenon, but rather comprises various subcultures and practices, each associated with different risks [31]. However, scientific literature has treated chemsex as a singular entity, overlooking significant differences beyond the types of substances used [12-19,21-23]. This oversight complicates the development of tailored health promotion programs for different user profiles within the chemsex community.

One possible quantitative approach to address this heterogeneity is to identify different session typologies through latent class analysis (LCA), which enables a person-centered approach. LCA has been used to analyze substance use typologies among SMM, often identifying a class with higher consumption of substances related to chemsex and referring to that group as chemsex users [32-36]. Other studies identify classes based on the number of substances that participants have used [37,38], focus only on slamming [39], or introduce sexualized substance use as one of the latent class indicators [40,41]. To the best of our knowledge, no LCA has been conducted to identify the different chemsex session classes that have been identified through qualitative research [31].

Thus, our study aims to estimate the prevalence of chemsex in a large and diverse sample, understanding chemsex sessions as a cultural phenomenon rather than just sexualized substance use, and to explore associated sociodemographic factors. Additionally, we seek to analyze distinct session classes with potential risk differences and the sociodemographic factors associated with these typologies.

## Methods

### Study Design and Population

A cross-sectional study was carried out using data from 5711 participants of an open web-based survey on substance use among SMM residing in Spain and aged 16 years or older.

Recruitment took place between May and July of 2020. Participants were mostly recruited through advertisements displayed or sent to all users on dating apps commonly used by the SMM community in Spain (Grindr, Scruff, Wapo, Bakala, MachoBB, GROWLr, and Xtudr), or via open invitations by LGBTQAI+ influencers, content creators, and key individuals on social media platforms (using YouTube videos and Instagram stories and posts). Additionally, participants were recruited in collaboration with LGBTQAI+ community organizations that sent invitations to their members and posted advertisements on their social media platforms.

The survey was anonymous and featured a self-administered questionnaire, thus reducing social-desirability bias and increasing both diversity and geographic variability within the sample [42,43]. No incentives were used. An IP check was carried out by the questionnaire website to ensure there were no duplicate answers from the same participant. The usability and technical functionality of the survey were tested before fielding the questionnaire. Respondents were able to review and change their answers before submitting the questionnaire.

The survey was designed and reported in accordance with the Checklist for Reporting Results of Internet E-Surveys (CHERRIES) [44].

### Measures

The questionnaire asked participants about any previous engagement in chemsex sessions with others, defined as using drugs during sex sessions in order to have specific sexual activities. In addition, it included a set of questions characterizing the sessions: the length of the sessions, the number of people that participants had condomless anal sex with during a session (both receptive and penetrative), intravenous use of substances in this context (slamming), and sexualized use of certain substances particularly related to chemsex and sexualized use (GHB, methamphetamine, mephedrone, ketamine, and poppers).

Additionally, the survey included questions regarding a set of sociodemographic variables: their age and migratory status, size of municipality of residence (rural areas with <50,000 inhabitants, medium-size cities with between 50,000 and 500,000 inhabitants, and big cities with >500,000 inhabitants), whether they considered their own financial situation to be comfortable, whether they were openly living their sexuality, whether they were living with HIV, and their lifetime steroid use.

### Data Analysis

The proportion of SMM that had engaged in chemsex sessions was calculated with a 95% CI. In order to estimate the sociodemographic factors associated with engaging in chemsex sessions, a multivariate Poisson regression with robust variance model was used to calculate adjusted prevalence ratios, along with the corresponding 95% CI. Prevalence ratios were adjusted in the regression by all the sociodemographic factors specified in the previous section.

We took a person-centered approach to the analysis; LCA was performed to identify and characterize meaningful latent classes or subgroups of participants that engaged in different kinds of chemsex sessions with different risk patterns [45]. We constructed dichotomous variables that characterized the sessions as latent class indicators: engaging in long sessions that last 6 hours or more; having condomless anal sex with 5 or more people in the same session; slamming; and sexualized use of GHB, methamphetamine, mephedrone, ketamine, or poppers. The same sociodemographic covariates used to examine association with engaging in sessions were included in the LCA to analyze their association with latent class membership. Only participants that engaged in chemsex sessions were included in the LCA. For the estimation of each model, we fixed a maximum of 20 repetitions with different sets of random starting values and 300 iterations per repetition. Models with 1-4 latent classes were fitted. To select the final model among those with different numbers of latent classes, we based our decision on a combination of goodness-of-fit statistical criteria (ll, Akaike’s information criterion, and Bayesian information criterion), parsimony, and interpretability.

All calculations were performed using Stata (version 17; StataCorp). Statistical significance was set at *P*<.05 for all calculations. Missing data were treated as such for all calculations.

### Ethical Considerations

All participants gave their informed consent to take part in this research project, which was approved by the Research Ethics Committee of the Institute of Health Carlos III (CEI-PI35_2020-v3). Privacy and confidentiality were ensured, as the survey was anonymous. No incentives or compensation were offered to participants. The importance of conducting the research from a nonstigmatizing perspective against the SMM and chemsex communities was considered throughout the whole process.

## Results

### Sociodemographic Characteristics and Chemsex Prevalence

Approximately half (3162/5711) of the participants were younger than 40 years. The vast majority of SMM in the sample were born in Spain (4717/5699), were not living with HIV (4041/5711), and had never used steroids (5303/5508). In addition, 43.3% (2369/5470) of participants lived in big cities, one-third (1713/5470) lived in medium-size cities, and one-quarter (1388/5470) lived in rural areas. Approximately two-thirds (3542/5477) of participants had a comfortable financial situation and 54% (3102/5709) openly lived their sexuality (Table 1).

The proportion of SMM engaging in chemsex sessions was 21.1% (1205/5711 participants; 95% CI 20%‐22.1%). Several sociodemographic factors were significantly associated with engaging in chemsex sessions: being a migrant (*P*=.04), not having a comfortable financial situation (*P*=.006), openly living their sexuality (*P*=.002), residing in bigger municipalities (*P*<.001), older age (*P*<.001), using steroids (*P*<.001), and living with HIV (*P*<.001), with adjusted prevalence ratios ranging from 1.17 to 2.01 (Table 2).

**Table 1.** Distribution of sociodemographic characteristics in a sample of sexual minority men derived from a cross-sectional survey carried out in 2020 in Spain (N=5711). Missing values for all variables are <0.25%, except for size of municipality (4.2%), financial situation (4.1%), and steroid use (3.6%).

	Values, n (%)	
**Age group, years (n=5711)**		
16‐29	1602 (28.1)	
30‐39	1560 (27.3)	
≥40	2549 (44.6)	
**Migratory status (n=5699)**		
Born in Spain	4717 (82.8)	
Migrant	982 (17.2)	
**Size of municipality of residence (n=5470)**		
<50,000	1388 (25.4)	
50,000‐500,000	1713 (31.3)	
>500,000	2369 (43.3)	
**Financial situation (n=5477)**		
Comfortable	3542 (64.7)	
Not comfortable	1935 (35.3)	
**Openly living their sexuality (n=5709)**		
No	2607 (45.7)	
Yes	3102 (54.3)	
**Living with HIV (n=5711)**		
No (HIV-negative)	4041 (70.8)	
Yes (HIV-positive)	794 (13.9)	
Unknown (never tested)	876 (15.3)	
**Steroid use, ever (n=5508)**		
No	5303 (96.3)	
Yes	205 (3.7)	

**Table 2.** Sociodemographic factors associated with engaging in chemsex sessions in a cross-sectional sample of sexual minority men from a survey conducted in 2020 in Spain.

	Adjusted prevalence ratio (95% CI)	
**Age group (years)**		
16‐29	Ref^a^	
30‐39	1.34 (1.12‐1.59)	
≥40	1.40 (1.19‐1.65)	
**Migratory status**		
Born in Spain	Ref	
Migrant	1.17 (1.01‐1.35)	
**Size of municipality of residence**		
<50,000	Ref	
50,000‐500,000	1.11 (0.93‐1.32)	
>500,000	1.33 (1.14‐1.56)	
**Financial situation**		
Comfortable	Ref	
Not comfortable	1.18 (1.05‐1.34)	
**Openly living their sexuality**		
No	Ref	
Yes	1.21 (1.07‐1.37)	
**Living with HIV**		
No (HIV-negative)	Ref	
Yes (HIV-positive)	2.01 (1.75‐2.30)	
Unknown (never tested)	0.59 (0.46‐0.74)	
**Steroid use, ever**		
No	Ref	
Yes	1.84 (1.49‐2.27)	

a Ref: reference category.

### Chemsex Session Typologies

**Figure 1.**
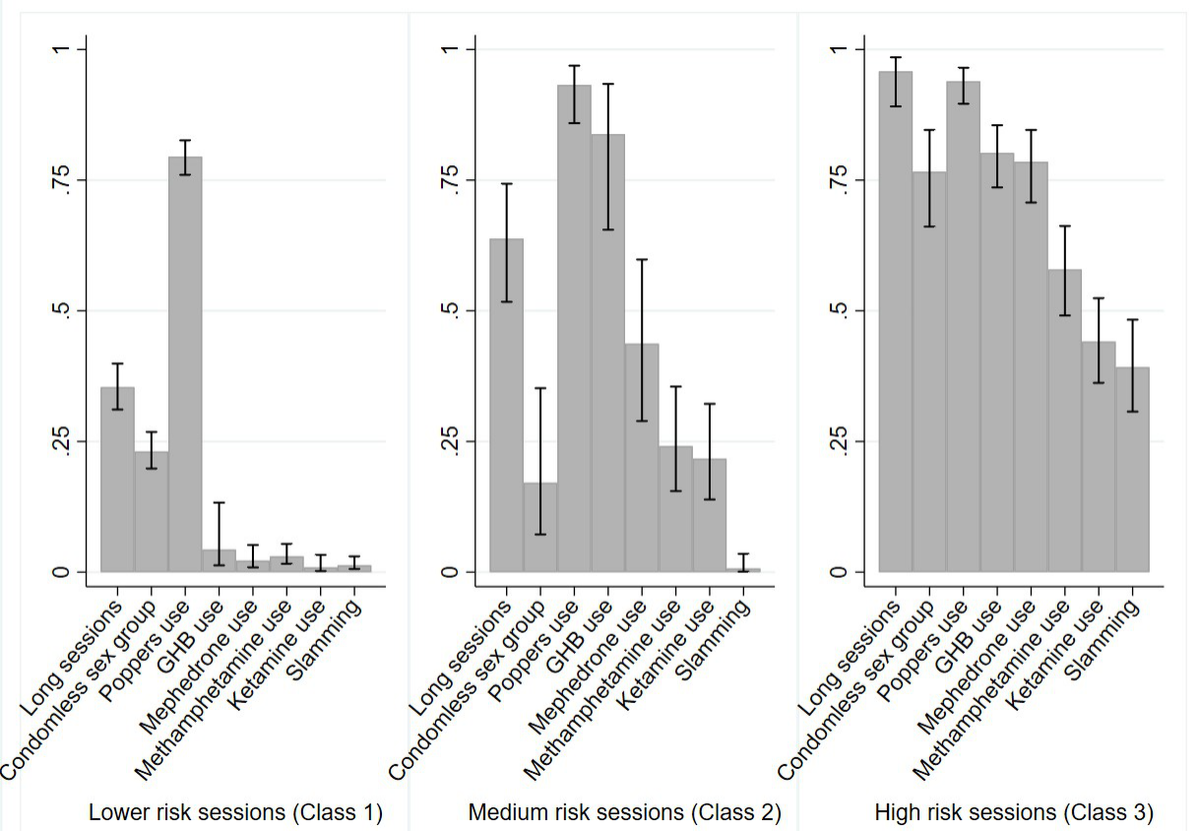
Chemsex sessions typologies conditional probabilities of risk behaviors and drug use within the latent classes in an online sexual minority men cross-sectional sample in 2020 in Spain.

A 3-class model was chosen considering a combination of goodness-of-fit statistical criteria (Multimedia Appendix 1), parsimony, and interpretability. The 3 latent classes identified are shown in Table 3 and Figure 1.

**Table 3.** Prevalence of chemsex session typologies and conditional probabilities of risk behaviors and drug use within the latent classes in a cross-sectional sample of sexual minority men from a survey conducted in 2020 in Spain. There were no missing values for substance use variables. Missing values were 2.3% for long sessions, 2.6% for condomless sex in a group, and 4.5% for slamming.

	Lower-risk sessions (class 1), percentage (95% CI)		Medium-risk sessions (class 2), percentage (95% CI)		Higher-risk sessions (class 3), percentage (95% CI)	
Class prevalence	57.2 (51.3‐62.9)		19.6 (13.7‐27.4)		23.2 (17.9‐29.6)	
**Risk behaviors and drug use**						
Long sessions^a^	35.4 (31.1‐39.9)		63.8 (51.7‐74.3)		95.8 (89.1‐98.5)	
Condomless sex in a group^b^	23.1 (19.8‐26.8)		17.1 (7.2‐35.2)		76.6 (66.1‐84.6)	
Poppers use	79.5 (76.0‐82.6)		93.2 (85.9‐96.9)		93.9 (89.6‐96.5)	
GHB^c^ use	4.3 (1.3‐13.3)		83.8 (65.5‐93.4)		80.2 (73.6‐85.5)	
Mephedrone use	2.2 (0.9‐5.2)		43.7 (28.9‐59.8)		78.5 (70.7‐84.6)	
Methamphetamine use	3.0 (1.6‐5.4)		24.1 (15.5‐35.5)		57.9 (49.1‐66.2)	
Ketamine use	0.9 (0.2‐3.3)		21.7 (13.9‐32.2)		44.1 (36.2‐52.4)	
Slamming	1.3 (0.6‐3.0)		0.7 (0.1‐3.5)		39.2 (30.7‐48.3)	

a Chemsex sessions lasting 6 hours or more.

b With 5 or more people in the same session.

c GHB: gamma-hydroxybutyrate.

Lower-risk sessions (class 1) represented 57.2% of chemsex participants. Fewer participants engaged in long sessions (35.4%) or condomless anal sex in groups (23.1%) compared to the other typologies. All substance use percentages were under 3% except for GHB (4.3%) and poppers (79.5%). There was a very low conditional probability of slamming (1.3%) in this class.

Medium-risk sessions (class 2) represented 19.6% of participants that engage in chemsex sessions. This group had a higher conditional probability of engaging in long sessions (63.8%) but a low probability of engaging in condomless anal sex in groups (17.1%). Use of all substances was over 20%, with higher use of mephedrone (43.7%), GHB (83.8%), and poppers (93.2%). This group had a very low probability of slamming (0.7%).

Higher-risk sessions (class 3) represented 23.2% of participants that engage in chemsex sessions. This group had a very high conditional probability of engaging in long sessions (95.8%) and condomless anal sex in groups (76.6%). The conditional probability of using all of the listed substances was over 40%, with higher use of mephedrone (78.5%), GHB (80.2%), and poppers (93.9%). This group had a slamming prevalence of 39.2%.

### Sociodemographic Factors Associated With Chemsex Typologies

First, we compared class 2 (medium-risk group) to class 1 (lower-risk group). For class 2, older participants had a lower probability of membership than participants aged 16‐29 years (odds ratio [OR] 0.54, 95% CI 0.31‐0.95) compared to membership in class 1. Probability of membership in class 2 was also lower among SMM with unknown HIV status compared to those that know they do not live with HIV (OR 0.33, 95% CI 0.17‐0.92), and was higher among SMM that openly live their sexuality compared to those who do not (OR 1.56, 95% CI 1.01‐2.42), relative to membership in class 1. The differences between the remaining sociodemographic variables were not statistically significant when comparing lower-risk and medium-risk classes (all *P* values >.05).

Next, we compared class 3 (higher-risk group) to class 1 (lower-risk group). The probability of membership in class 3 relative to membership in class 1 was lower for SMM aged over 40 years compared to those aged under 30 years (OR 0.55, 95% CI 0.33‐0.93). It was higher for participants living in bigger municipalities (OR 2.75, 95% CI 1.51‐5.02), using steroids (OR 2.78, 95% CI 1.57‐4.9), openly living their sexuality (OR 2.81, 95% CI 1.78‐4.43), and living with HIV (OR 4.99, 95% CI 3.20‐7.79). Migratory status and financial situation (*P* values >.05) were not significantly associated with latent class membership when comparing class 3 to class 1 (Table 4).

**Table 4.** Sociodemographic factors as predictors of latent class membership compared to the lower-risk session class (class 1) in a cross-sectional sample of sexual minority men from a survey conducted in 2020 in Spain.

	Medium-risk class (class 2) versus lower-risk class (class 1), odds ratio (95% CI)		Higher-risk class (class 3) versus lower-risk class (class 1), odds ratio (95% CI)	
**Age group (years)**				
16‐29	Ref^a^		Ref	
30‐39	0.87 (0.49‐1.52)		0.83 (0.48‐1.43)	
≥40	0.54 (0.31‐0.95)		0.55 (0.33‐0.93)	
**Migratory status**				
Born in Spain	Ref		Ref	
Migrant	0.64 (0.35‐1.17)		1.08 (0.71‐1.65)	
**Size of municipality of residence**				
<50,000	Ref		Ref	
50,000‐500,000	0.71 (0.37‐1.39)		1.90 (0.98‐3.68)	
>500,000	1.42 (0.84‐2.41)		2.75 (1.51‐5.02)	
**Financial situation**				
Comfortable	Ref		Ref	
Not comfortable	0.97 (0.63‐1.48)		0.81 (0.55‐1.19)	
**Openly living their sexuality**				
No	Ref		Ref	
Yes	1.56 (1.01‐2.42)		2.81 (1.78‐4.43)	
**Living with HIV**				
No (HIV-negative)	Ref		Ref	
Yes (HIV-positive)	1.41 (0.84‐2.36)		4.99 (3.20‐7.79)	
Unknown (never tested)	0.33 (0.17‐0.92)		0.61 (0.21‐1.76)	
**Steroid use, ever**				
No	Ref		Ref	
Yes	1.15 (0.48‐2.73)		2.78 (1.57‐4.93)	

a Ref: reference category.

## Discussion

### Principal Findings

The proportion of SMM that participated in chemsex sessions during their lifetime as reported in this study falls at the lower end of the range (17%-33%) reported by the vast majority of previously published papers [12-15,17-20,22,23,46]. However, this comparison is limited by the variability in chemsex definitions and the time frames used. Additionally, those previous studies did not consider the cultural component of chemsex sessions, focusing on any sexualized or nonsexualized use of certain substances to define chemsex, which may have resulted in overestimation of the proportion of SMM who participate in chemsex.

The LCA revealed that there are different typologies of chemsex participants, with distinct prevalence and behaviors that entail varying levels of risk, as qualitative evidence had previously indicated [31]. Previous research treated chemsex as a uniform phenomenon, at most differentiating based on the substances used. The proportion of SMM in the chemsex community participating in each type of session had not been studied before. These differences are particularly relevant considering the potential health consequences of chemsex—on both physical and mental health—including abscesses and other infections, overdoses, dangerous combinations of substances, drug side effects (renal, cardiac, etc), psychotic symptoms, or substance use disorder.

In our study, more than half of chemsex participants engaged in short sessions, characterized by infrequent condomless anal sex with multiple partners, low consumption of high-risk substances, and minimal slamming. Consequently, this group has a lower likelihood of engaging in problematic chemsex and experiencing associated negative consequences. The large proportion of SMM participating in chemsex sessions without using GHB, mephedrone, methamphetamine, or ketamine underscores the limitations of defining chemsex solely based on the consumption of these substances, rather than considering it as a cultural phenomenon within a community.

Approximately one-fifth of SMM engaging in chemsex participated in longer sessions with high GHB consumption, but where condomless anal sex with many partners remained infrequent, and slamming was almost anecdotal. Therefore, there is a group of chemsex participants that would benefit primarily from risk reduction strategies related to GHB use (eg, drug testing, starting with low doses and waiting before increasing, not mixing with other depressant drugs, recognizing and responding to signs of overdose, considering interactions with HIV medication).

Finally, about one-quarter of SMM in the chemsex community—and about 5% of all surveyed SMM—engaged in longer sessions with very high risks due to condomless anal sex with many partners, the substances used, and a high proportion of participants engaging in slamming. This is the group where health promotion and risk reduction strategies would be most effective, as problematic chemsex and negative health consequences are more likely to occur.

Chemsex participation varied across sociodemographic groups of SMM in our study, which should be considered when developing public health policies. As previous studies had found, the proportion of SMM who had engaged in chemsex was higher among older men [25-27,29,38], those living with HIV [23,25,29,38], those living in larger cities [25], and those open about their sexuality [25]. These associations should not be interpreted as if those factors were risk factors for engaging in chemsex sessions, as the correlation is bidirectional. Some behaviors that may occur in chemsex sessions, such as condomless anal sex with multiple partners (though the use of pre-exposure prophylaxis should also be considered) and slamming, increase the risk of HIV infection. Furthermore, as chemsex is inseparable from its cultural dimension, it is easy to understand how it is a more prominent phenomenon in bigger cities with larger queer communities and more common among men who are openly members of the community. It is important to interpret this from a nonstigmatizing perspective, considering the significant well-being, social, and health benefits of being involved in the community [47], and avoiding moral judgments from outside the culture, as well as avoiding paternalistic attitudes from public health officials and agencies.

Some previous studies have reported an increased probability of engaging in chemsex among migrant SMM [29,30], as found in our study, while others have not [25]. In contrast to a previous paper that described a higher chemsex rate among high-income SMM in a small sample [30], we found a greater probability of engaging in chemsex among those who are not in a comfortable financial situation. This can be explained by the higher drug use in lower-income social groups due to discrimination and structural sociodemographic factors that result in poorer health outcomes in those groups and the potential financial consequences of problematic drug use. Another study had noted more common use among SMM with higher educational levels [25]. However, it could be argued that educational level may not serve as a suitable proxy for financial situation within the LGBTQAI+ community, as it does for cis heterosexual men, due to employment discrimination [48].

To the best of our knowledge, no previous studies have conducted a multivariable analysis to examine the relationship between anabolic steroid use and chemsex. In our study, we identified a higher probability of engaging in chemsex among SMM who use steroids. This association has been observed previously in other drug use contexts [49], and it can be explained within the chemsex community by its link to masculinity roles and body image pressure among SMM [50]. In light of these findings, public health policy makers should consider the impact of masculinity perceptions and fatphobia in the LGBTQAI+ community when formulating chemsex policies.

The same sociodemographic factors associated with engaging in chemsex among SMM are also linked to participating in higher-risk sessions compared to lower-risk sessions, with the exception of financial situation and age. Older SMM were more likely to have engaged in chemsex but less likely to participate in higher-risk sessions. Notably, living with HIV was particularly associated with engaging in those high-risk sessions, which could be explained by prevalent behaviors in those sessions that increase the risk of HIV infection, even though the vast majority of SMM living with HIV in Spain have an undetectable and therefore untransmittable viral load [51]. Additionally, HIV stigma can contribute to drug use among people living with HIV. Even though correlation is bidirectional, this underscores the importance of recognizing that chemsex and its riskier variations are not a matter of individual choices, as some neoliberal frameworks may suggest. Instead, chemsex constitutes a cultural phenomenon profoundly influenced by social structures. Therefore, public health professionals must consider these broader sociodemographic factors when developing policies and health promotion strategies targeting both chemsex in general and higher-risk session harm reduction, while also considering how the criminalization of drug users increases risks and harm [52,53].

This study is subject to certain limitations. A nonprobabilistic sampling method was used, which may have resulted in some bias, as participants were SMM present on dating apps or active on social networks and following LGBTQAI+ influencers or activist community organizations. This could limit the generalizability and external validity of the results, which may differ in other countries. However, it is worth noting that, despite the sensitivity of the topic, different recruitment settings were used to decrease bias and increase generalizability. The web-based sampling approach allowed for a much larger sample of SMM and increased geographic variability and diversity [42,43]. This study did not distinguish between problematic and nonproblematic chemsex, which should be addressed in future research, as well as polysubstance use and drug mixtures that could increase risks. Other sociodemographic variables that could contribute to a more comprehensive understanding of chemsex from a cultural perspective were not measured, such as perceptions of masculinity, body image pressure, or internalized stigma. Exploring these aspects in future research may provide valuable information about the cultural component of chemsex. Future research should also focus on how chemsex varies in different contexts. The analysis was not preregistered and the results should be considered exploratory.

On the other hand, this study also possesses important strengths. First, it included one of the largest SMM samples to date. Our web-based sampling method allowed for a richer representation of the community than samplings in sexually transmitted infection clinics or those that only included SMM living with or without HIV. Additionally, we studied chemsex while considering it as a cultural phenomenon and not merely the sexualized use of certain substances. Understanding the cultural dimension of chemsex is fundamental to advancing research and policies that are useful for the SMM community. Furthermore, to the best of our knowledge, this is the first quantitative study that explores different forms of chemsex sessions and their varying health potential risks using LCA.

### Conclusions

Chemsex is relatively common among SMM in Spain. Nevertheless, despite the tendency to view chemsex as a uniform phenomenon, there are different typologies of sessions, and the proportion of SMM engaging in high-risk sessions is low. Consequently, chemsex should become a public health priority, and health institutions should reinforce their chemsex-related health promotion programs using a nonstigmatizing approach. However, the fact that there are different forms of sessions with varying risks and prevalence has to be considered by those programs.

Additionally, chemsex is highly related to sociodemographic factors. It is noteworthy that the sociodemographic factors associated with participation in chemsex may not necessarily align with those associated with engaging in higher-risk sessions. This information should be duly considered in chemsex policies and strategies. The association between chemsex and some sociodemographic factors further underscores the cultural dimension inherent in chemsex, which should be considered in both comprehensive research and health promotion programs.

## Supplementary material

10.2196/60012 Multimedia Appendix 1 Latent class analysis goodness-of-fit statistical criteria.

## Abbreviations

CHERRIES: Checklist for Reporting Results of Internet E-Surveys
GHB: gamma-hydroxybutyrate
LCA: latent class analysis
OR: odds ratio
SMM: sexual minority men
