# Supplementary material for: Chemsex Session Typologies and Associated Sociodemographic Factors in Sexual Minority Men: Latent Class Analysis From a Cultural Perspective Using a Cross-Sectional Survey
Source: JMIR Public Health Surveill. 2024 Sep 27;10:e60012. doi: 10.2196/60012 (PMC11451521; doi:10.2196/60012)
Supplement: Multimedia Appendix 1 [file publichealth-v10-e60012-s001.docx]

**Supplementary Table S1**. Latent class analysis goodness of fit statistical criteria

| **Number of classes** | $\mathcal{ll}$ | **AIC** | **BIC** |
| --- | --- | --- | --- |
| 1 | -4801.4 | 9618.8 | 9659.0 |
| 2 | -4054.6 | 8163.1 | 8299.0 |
| 3 | -3988.9 | 8069.7 | 8301.2 |
| 4 | -3936.8 | 8003.6 | 8330.7 |
